# Functional Source Separation for EEG-fMRI Fusion: Application to Steady-State Visual Evoked Potentials

**DOI:** 10.3389/fnbot.2019.00024

**Published:** 2019-05-14

**Authors:** Hong Ji, Badong Chen, Nathan M. Petro, Zejian Yuan, Nanning Zheng, Andreas Keil

**Affiliations:** ^1^Department of Automation Science and Technology, Institute of Artificial Intelligence and Robotics, Xi'an Jiaotong University, Xi'an, China; ^2^Department of Psychology, Center for Brain, Biology, and Behavior, University of Nebraska-Lincoln, Lincoln, NE, United States; ^3^Department of Psychology, Center for the Study of Emotion and Attention, University of Florida, Gainesville, FL, United States

**Keywords:** simultaneous EEG and fMRI, steady-state visual evoked potentials (ssVEP), functional source separation (FSS), simulated annealing (SA), aversive conditioning

## Abstract

Neurorobotics is one of the most ambitious fields in robotics, driving integration of interdisciplinary data and knowledge. One of the most productive areas of interdisciplinary research in this area has been the implementation of biologically-inspired mechanisms in the development of autonomous systems. Specifically, enabling such systems to display adaptive behavior such as learning from good and bad outcomes, has been achieved by quantifying and understanding the neural mechanisms of the brain networks mediating adaptive behaviors in humans and animals. For example, associative learning from aversive or dangerous outcomes is crucial for an autonomous system, to avoid dangerous situations in the future. A body of neuroscience research has suggested that the neurocomputations in the human brain during associative learning involve re-shaping of sensory responses. The nature of these adaptive changes in sensory processing during learning however are not yet well enough understood to be readily implemented into on-board algorithms for robotics application. Toward this overall goal, we record the simultaneous electroencephalogram (EEG) and functional magnetic resonance imaging (fMRI), characterizing one candidate mechanism, i.e., large-scale brain oscillations. The present report examines the use of Functional Source Separation (FSS) as an optimization step in EEG-fMRI fusion that harnesses timing information to constrain the solutions that satisfy physiological assumptions. We applied this approach to the voxel-wise correlation of steady-state visual evoked potential (ssVEP) amplitude and blood oxygen level-dependent imaging (BOLD), across both time series. The results showed the benefit of FSS for the extraction of robust ssVEP signals during simultaneous EEG-fMRI recordings. Applied to data from a 3-phase aversive conditioning paradigm, the correlation maps across the three phases (habituation, acquisition, extinction) show converging results, notably major overlapping areas in both primary and extended visual cortical regions, including calcarine sulcus, lingual cortex, and cuneus. In addition, during the acquisition phase when aversive learning occurs, we observed additional correlations between ssVEP and BOLD in the anterior cingulate cortex (ACC) as well as the precuneus and superior temporal gyrus.

## 1. Introduction

An organism's survival depends on its ability to quickly and adaptively respond to environmental challenges and opportunities. To accomplish this task, the mammalian brain detects and stores the predictive value of recurring environmental signals with respect to dangerous or rewarding outcomes (Sokolov, 1963). Established associative networks that link specific stimuli to representations of biological significance and motor action are the mutual interest to Neuroscientists and Neurorobotists (Falotico et al., 2017; Oess et al., 2017). An extensive literature has demonstrated that the neural and hemodynamic amplification of threat, relative to neutral, cues is paralleled by a host of behavioral effects such as facilitated detection (Öhman and Soares, 1998), identification (Anderson, 2005), and greater perceptual vividness (Todd and Thompson, 2015).

Hemodynamic and electrophysiological time series represent the two major measurement modalities used in cognitive neuroscience research today. They convey complementary, unique information, with each modality possessing sensitivity to different facets of brain physiology. Above and beyond their well-established differences in temporal and spatial resolution, fMRI-BOLD signals, and electrical field potentials often display divergent responses to experimental manipulations, even in situations where they are considered indices of the same large-scale neural process. Thus, characterizing the relationship between hemodynamic and electrophysiological time series has important implications for neurophysiological studies of the human brain. Consistent with recent empirical studies into the nature of the BOLD signal (Logothetis et al., 2001; Logothetis, 2015), and with the biophysics of EEG (Nunez and Srinivasan, 2006), the EEG-fMRI fusion approach examined in the present study assumes that co-variation between EEG and fMRI signals accounts only for fraction of the variance of each measure (Herrmann and Debener, 2008). To facilitate cross-validation and precise prediction of expected patterns of co-variation, we use non-invasive simultaneous EEG-fMRI recordings during a fear conditioning task in which strong and sustained visuocortical responses are elicited. Specifically, we combine fMRI with steady-state visual evoked potentials (ssVEPs), elicited by stimuli that are rapidly and periodically modulated in luminance or contrast. Using standard psychophysiological techniques such as EEG, these responsing signals can be recorded at the surface of the scalp as an oscillatory waveform that has the same fundamental frequency as the driving stimulus (Regan, 1989). Readily quantified at the level of single trials and of known origin in extended visual cortical areas, this approach allows us to capture different aspects of co-variation in simultaneously EEG and fMRI (Liu et al., 2012): the correlation may reflect responses to experimental events seen in both modalities, mixing trivial effects of joint reactivity of EEG and fMRI to salient external events with correlations of interest such as condition-specific co-variation, or co-variation with various cognitive states. In addition, and of particular relevance in the absence of experimental stimulation, specific portions of the co-variance between modalities may reflect neural-hemodynamic dependencies in spontaneous, ongoing brain activity. In previous work, we have described a method for quantifying the spatial and temporal correlations between the time series of fMRI BOLD and suitable indices derived from the EEG signal (Ji et al., 2018). The present work examines in more detail the benefits of ssVEP preprocessing using Functional Source Separation (FSS) as an optimization step.

**Figure 1:**
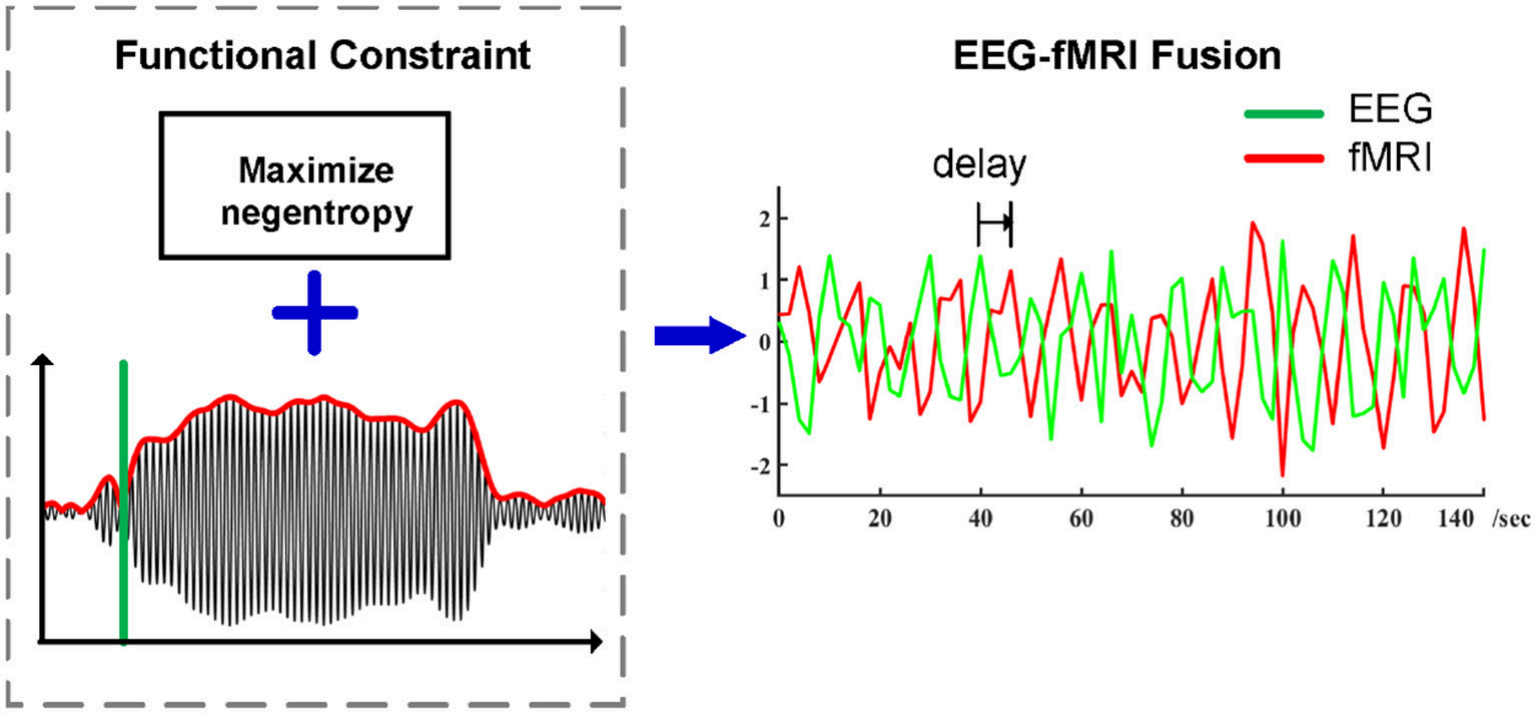
Preprocessing pipeline of ssVEP-fMRI fusion using Functional Source Separation (FSS) as an optimization step, timing information which enhances the identification and separation of relevant signals in electrophysiological time series while maximizing negentropy constraint in the meantime, the summarized ssVEP time series then go for the spatial-temporal correlation analysis with a proper temporal delay.

The ssVEP possesses a high signal-to-noise ratio in addition to other desirable qualities for EEG-fMRI fusion: The frequency of the ssVEP is precisely known and can therefore be reliably separated from noise and quantified in the frequency domain (Spekreijse et al., 1985; Regan, 1989; Wang et al., 2007). The high signal to noise ratio of ssVEPs allows the robust quantification of visual neuronal population responses at the level of individual trials, of particular interest in the context of EEG-fMRI studies. In terms of neurocognitive function, fluctuations in ssVEP amplitude are sensitive to stimulus content (e.g., Keil et al., 2003) as well as task instructions (e.g., Müller et al., 1998; Attar et al., 2010). Generators of the ssVEP are in the extended visual cortex (including occipital, temporal, and parietal cortices; Müller et al., 1997), with most pronounced contributions from retinotopic areas (Wieser and Keil, 2011), but also from cortices higher in the visual hierarchy, along the ventral and dorsal streams (Di Russo et al., 2007). The ssVEP technique thus represents a robust and reliable method for non-invasively isolating population-level neuronal responses at low levels of the traditional visual hierarchy (Spekreijse et al., 1985; Regan, 1989), very well-suited for joint validation of EEG-fMRI analyses, in which it has been previously used (Sammer et al., 2005). Existing pipeline for ssVEP-fMRI analyses have shown high variability across participants and surprisingly small co-variation of ssVEP amplitude and BOLD magnitude in visual areas. Given that flickering or contrast-reversing stimuli evoke very strong BOLD and electrophysiological responses, it is conceivable that previous findings reflect noise in the single-trial estimation of ssVEPs rather than a lack of co-variation. The present study illustrates the application of a new preprocessing pipeline for ssVEP-fMRI fusion and presents its outcomes regarding group-level findings, including the correspondence of ssVEP-fMRI covariation maps among individual participants (see Figure 1).

EEG quality is a crucial issue when conducting joint EEG-fMRI analyses. Blind source separation (BSS) represents a group of techniques for extracting signals in presence of noise, by exploiting the statistical properties of sources. For example, independent component analysis (ICA) recovers independent sources that contribute to form the measured signal, under the assumptions that they are linearly mixed, and measured with an array of spatially distributed sensors (Comon, 1994; Bell and Sejnowski, 1995; James and Hesse, 2004). ICA is widely used in neurophysiological time series analysis (Makeig et al., 2002; Delorme and Makeig, 2004) and is increasingly the standard method when performing single-trial based EEG-fMRI fusion (Debener et al., 2005; Eichele et al., 2005; Bénar et al., 2007; Mulert et al., 2008) following removal of gross MRI artifacts. It has also been successfully used for removal of eye blinks, eye movements, and electrode artifacts (Debener et al., 2006; Mantini et al., 2007), denoising muscle artifacts (Chen et al., 2017, 2018) and MR noises (Acharjee et al., 2015). Several novel techniques have been proposed for multiset and multimodal fusion analysis (Chen et al., 2013, 2016). Instead of an entirely blind searching approach that relies purely on statistical properties of the signals, the current study used Functional Source Separation (FSS). This method uses timing information to constrain the solutions, that satisfy physiological assumptions. FSS thus represents an optimization step in EEG-fMRI fusion, with the goal of improving the signal quality of EEG data recorded in the MRI environment. FSS is a semi-blind extension of ICA that incorporates prior knowledge about the responses of interest (Barbati et al., 2006). Thus, FSS enhances the identification and separation of relevant signals in electrophysiological time series while aiming to maximize the neurophysiological validity that is typically associated with using the averaged signal (Ostwald et al., 2010, 2011; Porcaro et al., 2010, 2011; Naik and Wang, 2014). In the present pipeline, following removal of gradient and cardioballsitic artifacts with standard techniques, data were further processed using FSS.

The present paper illustrates the usage of this FSS based approach for quantifying ssVEP-fMRI co-variation. Using a fusion algorithm that we have previously described (Ji et al., 2018), we applied the FSS preprocessing step to data from a 3-phase aversive conditioning paradigm that included a habituation, acquisition, and extinction phase. EEG and fMRI were recorded simultaneously while participants viewed periodically (10Hz) and rapidly phase-reversing Gabor patches (sine-wave gratings), evoking steady-state visual potentials (ssVEPs).

## 2. Materials and Methods

### 2.1. Participants

Participants consisted of 10 undergraduate and graduate students (4 male, average age 20.8 years) who, after giving written informed consent, participated on a volunteer basis or received course credit in the General Psychology course taught at the University of Florida. All participants were screened for metallic implants, claustrophobia, and history of seizure episodes. Female participants self-administered a pregnancy test prior to participation. Data of one additional subject was excluded from the analysis for poor quality of EEG data, defined as retaining <50% of the trials after artifact screening. All procedures were approved by the institutional review board of the University of Florida and were consistent with the Declaration of Helsinki on studies with human participants.

### 2.2. Stimuli and Experiment Setup

Stimuli were Gabor patches, i.e., sinusoidal gratings multiplied with a Gaussian envelope. They were oriented at either 15 or 345° relative to the vertical meridian. Gabor patches were shown at a maximum Michelson contrast of 95% (maximum = 110*cd*/*m*^2^; minimum = 2.1*cd*/*m*^2^) on a dark gray background, at a spatial frequency of 0.45 cycles per degree, and a horizontal visual angle of 15.5°. Participants viewed all stimuli on a mirror placed on the MR head-coil positioned 8.5 cm from eyes, allowing them to see an MR-compatible monitor that was placed outside the scanner bore. The stimuli reversed their phase every 100 ms to evoke a ssVEP having its second harmonic at 10 Hz. Consistent with previous studies of phase-reversal ssVEP (see Keil et al., 2013; Norcia et al., 2015 for a review), we analyzed the second harmonic response, i.e., 10 Hz.

The data presented in this report were recorded from a differential aversive conditioning study in which Gabors of one orientation (but not the Gabor patches with the other orientation) were occasionally paired with an electric shock (see Petro et al., 2017, for details). For the habituation block, participants were instructed that they would not feel any shock but to fixate on the patterns. During the acquisition block, participants were informed that they would intermittently feel a cutaneous electric shock during the experiment but were not instructed as to the contingencies of the shock administration. The extinction phase was also uninstructed, such that participants were not told that no more shocks were to be given. Each participant was instructed to remain still while in the scanner and to maintain fixation on the center of the screen. All stimulus presentation was controlled using E-Prime software (Psychology Software Tools, Pittsburgh, PA). Cutaneous shocks were administered using an STMISO Stimulation Isolation Adapter (BIOPAC Systems, Inc., Goleta, CA) with MRI-compatible skin electrodes.

The data reported here include 40 total trials per phase per participant. Each trial consisted of one of the two gratings being presented for 5,100 ms, during which its phase was alternated every 100 ms. An inter-trial interval (ITI) consisted of an initial gray cross (37.5*cd*/*m*^2^; 1° of visual angle) presented in the middle of the screen for a random duration between 0 and 8 s followed by a white cross (149.0 *cd*/*m*^2^) for a duration of 3 s, immediately preceding trial onset with Gabor patch presentation.

EEG data were recorded on a 32-channel MR compatible system (BrainAmp MR plus amplifier by BrainProducts). This system consisted of thirty-one Ag/AgCl electrodes placed on the head according to the 10–20 system and one cardioballistic (CB) electrode placed on the upper back to record heart-beats. The reference was positioned at FCz, the ground electrode was placed 1 cm anterior to Oz. Impedances were reduced to below 10 kΩ for all scalp electrodes and below 50 kΩ for the cardio electrode, as suggested by the Brain Products manual. According to the Brain Vision user manual, a sampling rate of 5 kHz EEG permits the TDD (Template Drift Detection) algorithm the highly accurate detection of drifts and shifts in typical MR data set, therefore EEG data were recorded online at 5 kHz and digitized to 16 bit, while the digitized data is transferred via a fiber-optic cable to the recording computer. The system was synchronized to the internal clock of the scanner, and synchronized event marker has been added for further signal processing.

MRI data were collected with a 3T Philips Achieva scanner. An Avotec Silent Scan headphone system was used to diminish gradient noise. Data were acquired during gradient-echo echo-planar imaging sequence [echo time (TE), 30 ms; repetition Time (TR), 1.98 s; flip angle, 80°; slice number, 36; field of view, 224 mm; voxel size, 3.5 × 3.5 × 3.5*mm*^3^; matrix size 64 × 64]. The first four functional scans were discarded to allow for scanner stabilization. Slices were acquired in ascending order oriented parallel to the plane connecting the anterior and posterior commissure during an 1,850 ms interval with 130 ms between each TR, during which no images were collected and allowed visual inspection of the EEG data during recording when the MR gradient artifact was absent. A T1-weighted high-resolution structural image was obtained after completion of all functional scans.

### 2.3. Artifact Handling and Preprocessing

Brain Vision Analyzer 2.0 (Brain Products) Software was used to remove artifacts caused by the magnetic gradients and heart-beats. Gradient artifacts are due to the switching of magnetic resonance (MR) gradients that is necessary to collect MRI data. The ballistocardiogram (BCG) artifact is related to the pulsatile movement of blood and the pulsatile movement of electrodes adjacent to large blood vessels. These two sources of noise are relatively independent. The removal of magnetic gradient artifacts followed a modified version of the algorithm developed in Allen et al. (2000), in which an artifact template constructed from a sliding window of 41 consecutive volumes is subtracted from the EEG data according to the event markers sent from the scanner's internal clock. Cardioballistic artifacts were removed with a standard algorithm as well (Allen et al., 1998). This method detected R peaks in the CB electrode and created a template from 21 consecutive heartbeats, which were subtracted from the continuous EEG data. Particular attention was given to reliable ECG peak detection: Misaligned and falsely detected R peak markers were corrected manually according to the peak of the correlation curve. Data was then down-sampled to 250 Hz. To isolate non-cerebral signals such as eye blinks, ICA was applied, and components with distinct peaks at sensors FP1 and FP2 removed (Delorme and Makeig, 2004). Then, the data were referenced to a common average, removing any global, non-cerebral signals that were common to all sensors.

Preprocessing of BOLD fMRI data was completed using SPM12 (Eickhoff et al., 2005, freely available at http://www.fil.ion.ucl.ac.uk/spm/). We followed the standard preprocessing routines suggested by SPM: Time differences were compensated by slice timing correction. Head movements were estimated by realigning each scan to match one representative scan with rigid transformation. Images were normalized and registered to the Montreal Neurological Institute (MNI) space. Functional volume images were re-sampled to a spatial resolution of 3 × 3 × 3 *mm*^3^. Images were smoothed using a Gaussian kernel with a full-width at half-maximum of 6 mm. Low-frequency temporal drifts were removed from the BOLD data using a 1/128 Hz high-pass filter.

### 2.4. FSS With Prior Information

**Figure 2:**
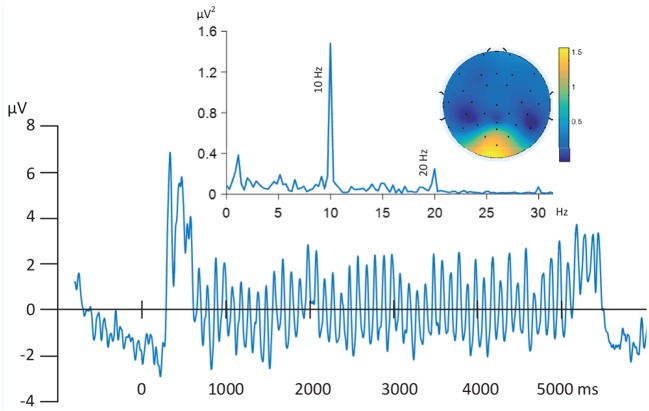
Grand Mean time series of filtered (0.5–30 Hz) EEG time-series (Oz) time locked to trial onset and averaged across all trials and subjects (bottom). Oscillatory activity driven by stimuli picture evoked flicker stream (10 Hz) are the dominant signals as illustrated by the frequency domain spectrum (top left), most strongly represented by posterior electrodes in the topographic map (top right).

Electroencephalography (EEG) reflects postsynaptic activity in large populations of cortical neurons that have similar spatial orientation (Nunez and Srinivasan, 2006). EEG signals are often assumed to be generated by a linear mixing process of many sources of activity (i.e., many such populations), which may overlap spatially and temporally and are distorted due to the effect of volume conduction. For the ssVEP, the known origin of the signal is located in lower-tier (calcarine fissure) and extended visual cortical areas. Because of the desirable features of the ssVEP (known frequency, high signal-to-noise ratio) robust ssVEP signals can be obtained in the fMRI scanner environment. As illustrated by Figure 2, the EEG epochs were extracted in the range of 1 s pre-stimulus and 6 s post-stimulus onset. The resulting data segments of 7 s were transformed into the frequency domain (top left). In the resulting spectrum, the maximum absolute value of the Fourier coefficients was observed at the driving frequency of 10 Hz, most pronounced at posterior electrodes (topographic map, top right). Furthermore, the Grand Mean time domain average across all trials and subjects shows enhanced evoked oscillatory activity (bottom) during the period when the phase-reversing stimulus was on, compared to baseline. FSS aims to maximize the neurophysiological validity that is associated with this latter property of the averaged signal. Therefore, we use timing information to constrain the solutions, while also considering the independence source constraint, which enhances the identification and separation of relevant signals in electrophysiological time series. The modified contrast function is:

(1) $$\begin{array}{r} F\left(\text{w}\right)=J\left(\text{w}\right)+\lambda H\left(\text{w}\right) \end{array}$$

The FSS optimization tends to find a projection vector **w** that maximizes the above contrast function. *J* can be any function for ICA. Here, we adopted the criteria of fastICA (Hyvarinen, 1999), aiming to minimize the Gaussianity of the results (equivalently, to find the separation vector in which the negentropy is maximized), since the observed mixed signals will tend to have more Gaussian amplitude distributions. The function *H* accounts for prior information we have on sources, which give preference to solutions that tend to satisfy physiological assumptions. Parameter λ is used to weigh the two parts of the contrast function, chosen to be 10 for ssVEP recovery.

The function *J* serves as a generalization of the cumulant-based approximation of negentropy

(2) $$\begin{array}{r} J\left(\text{w}\right)={\left[\text{E}\left\{\text{G}\left({\text{w}}^{\text{T}}\text{x}\right)\right\}-\text{E}\left\{\text{G}\left(\text{v}\right)\right\}\right]}^{2} \end{array}$$

where **x** is the pre-processed EEG and **v** is a standardized Gaussian variable. The variance of **w**^*T*^**x** must here be constrained to unity. The following choices of *G* have proven useful according to Hyvarinen (1999), Hyvärinen and Oja (2000)

(3) $$\begin{array}{r} G\left(u\right)=\frac{1}{a_{1}}log\left(cosh\left(a_{1}u\right)\right) \end{array}$$

where 1 ≤ *a*_1_ ≤ 2 is some suitable constant, we here chose *a*_1_ = 1.

To facilitate the FSS optimization, a zero-phase narrow bandpass filter centered at the target frequency 10 Hz with plus and minus 0.5 Hz was applied. The stimulus response is defined as

(4) $$\begin{array}{r} R_{stim}=\frac{1}{t_{e}-t_{s}}\int_{t_{s}}^{t_{e}}abs\left(Hilbert\left(EA\left(t\right)\right)\right)dt\\ -\frac{1}{0-t_{b}}\int_{t_{b}}^{0}abs\left(Hilbert\left(EA\left(t\right)\right)\right)dt \end{array}$$

with the evoked activity, EA, computed by averaging signal epochs of the estimated source **w**^*T*^**x**, which lasted for 5 s for the post-stimulus reactivity and 1 s for the pre-stimulus baseline. Accordingly, the upper and lower limits of the integral are set as *t*_*s*_ = 0, *t*_*e*_ = 5 and *t*_*b*_ = −1. The operation *abs*(*Hilbert*(·)) extracts the envelope of the evoked activity (Bracewell and Bracewell, 1986; Huang, 2014). Following the former work (Barbati et al., 2006) which apply FSS on MEG data, a saturation function is adopted in function *H* and tuned to suit the ssVEP study:

(5) $$\begin{array}{r} H=\psi \left(R_{stim},k\right) \end{array}$$

where

$$\psi (R_{stim},k)=\;\{\begin{array}{ll} R_{stim}/k & \text{when }R_{stim}\leq \text{k}\\ 1 & else \end{array}$$

where *k* is the parameter measuring the required minimum response, so as to define an admissible region where the optimization is only driven by *J* when response is greater than *k*. For the present application, the maximum *R*_*stim*_ for each phase is computed separately with fastICA, and assigned to *k*. Since prior information about the sources can only be described by a non-differentiable function, simulated annealing is employed for global optimization (Kirkpatrick et al., 1983; Ingber, 2000). To ensure the stability and reliability of the optimization procedure, the contrast function for all three phases were added up and jointly optimized.

The approach described above gives us the possibility to extract only one component that maximizes the functional behavior in agreement with the functional constraint.

### 2.5. Quantifying the SNR of Extracted ssVEP

In order to evaluate the performance of FSS optimization, the signal-to-noise ratio is determined by comparing the signal level during the stimuli on period with the signal level without stimulus. The pre-stimulus baseline from −1 to 0 s was set as the time interval of background/baseline noise, during which the participant was instructed to maintain fixation on a white cross, preceding the onset of the Gabor patch. In addition, a period from the inter-trial baseline (5.6–7 s after stimulus onset) was also included as a manipulation check, quantifying the extent to which the extracted ssVEP oscillation attenuates after stimulus offset. Since this part of background information is blind to FSS optimization, it represents an intuitive test for neurophysiological validity.

### 2.6. ssVEP-BOLD Fusion

To assess the spatial-temporal correlation between EEG and fMRI responses recorded simultaneously, the recovered ssVEP source was averaged across time segments of each fMRI scan, representing the intensity of neuronal activities, resulting in a time series with the same temporal resolution of the BOLD. As in the procedure of FSS optimization described in methods, the proper scaling (including sign) of the source signals is non-recoverable, we are mostly interested in the predictability reflects the proportion of the variance in the BOLD signal that is linearly predicted from ssVEP time series. Following the previous work (Wang and Zheng, 2014; Ji et al., 2018), we use the bivariate case of cross multivariate correlation coefficient (xMCC) for correlation analysis between EEG and fMRI, which is equal to the absolute value of the cross correlation coefficient. The metabolic (BOLD) events are slower and temporally lagged to the neuro-electric activity (EEG). Based on the large body of research on stimulus-BOLD latency performed in the visual cortex (Buxton et al., 1998; Serences, 2004; Penny et al., 2011), we adopt the consensus setting of 4 s as the latency of BOLD relative to neural events.

### 2.7. Statistical Inference: Permutation Testing

To assess the statistical significance of the correlation results, we applied permutation tests to determining the threshold for rejecting the null hypothesis of no linear relation based on shuffled data, and assumed statistical significance (permutation controlled in brain regions) if *p*-value is less than 0.05 for single participant. Given the small size of the present sample, the robustness of a given ssVEP-fMRI correlation across participants was quantified by measuring inter-subject overlap of voxels showing significant correlations after thresholding by a permutation test, using a binomial test. To this end, we calculated the probability of overlap at a given voxel under the null hypothesis that significant correlations occurred in different voxels and different participants. The resulting *p*-values were then corrected by the number of voxels considered overall, resulting in a threshold of 0.5 at a given voxel (i.e., five out of 10 participants showing permutation controlled, significant correlations at that voxel). These results were compared to the average correlation map, to establish the validity of this conservative statistical approach.

## 3. Results

### 3.1. EEG-ssVEP Source Extraction

The SNR index was evaluated for each participant and experimental phase separately, and compare with ICA results optimized for each phase separately. As a referencing index, in Table 1 we reported the spectrum signal-to-noise ratio (SNR_RAW), which divides the power of ssVEP at 10 Hz by the mean power of neighboring bins in the spectral range between 0.5 and 30 Hz, together with ssVEP amplitude (ssVEP_AMP) (Keil et al., 2008), by averaging the EEG traces in the moving windows in the time domain, which was then submitted to a Discrete Fourier Transform to get the 10 Hz ssVEP amplitude.

**Table 1:** SNR index for ICA and FSS optimization were calculated, for each participant and experimental phase.

**PHASE**	**Subj_ID**	**SNR_ICA**	**SNR_FSS**	**SNR_RAW**	**ssVEP_AMP**
HAB	Sub_01	6.67	7.59	10.96	2.62
	Sub_02	3.64	4.90	17.10	2.27
	Sub_03	3.79	7.30	19.49	3.23
	Sub_04	1.89	2.03	2.39	0.67
	Sub_05	5.07	8.60	14.77	2.79
	Sub_06	4.76	3.30	14.83	2.08
	Sub_07	2.09	1.68	3.40	0.97
	Sub_08	2.43	4.40	7.41	1.18
	Sub_09	3.28	5.18	6.17	1.28
	Sub_10	2.72	3.31	4.85	0.63
ACQ	Sub_01	5.91	6.29	12.30	2.91
	Sub_02	3.35	3.95	21.32	2.32
	Sub_03	7.66	7.39	17.53	2.79
	Sub_04	2.91	2.83	2.66	0.58
	Sub_05	8.50	9.01	19.34	2.78
	Sub_06	2.65	4.09	8.43	1.90
	Sub_07	1.28	2.27	2.26	1.63
	Sub_08	2.60	7.00	10.35	1.24
	Sub_09	2.47	10.27	4.67	0.96
	Sub_10	3.49	3.06	2.49	0.79
EXT	Sub_01	2.69	6.85	9.02	2.94
	Sub_02	4.68	3.34	16.48	2.10
	Sub_03	5.79	6.96	17.18	2.78
	Sub_04	1.11	1.92	1.35	0.67
	Sub_05	4.27	5.28	23.74	3.05
	Sub_06	2.02	4.45	8.60	1.67
	Sub_07	1.11	1.54	2.44	1.16
	Sub_08	4.33	4.55	10.85	1.27
	Sub_09	3.90	4.49	4.43	0.71
	Sub_10	2.13	2.96	6.92	0.89

*Raw spectrum SNR (sensor Oz, 0.5–30 Hz) and amplitude of ssVEP are given as a reference. SNR_FSS is underlined when SNR_FSS is lower than SNR_ICA*.

**Figure 3:**
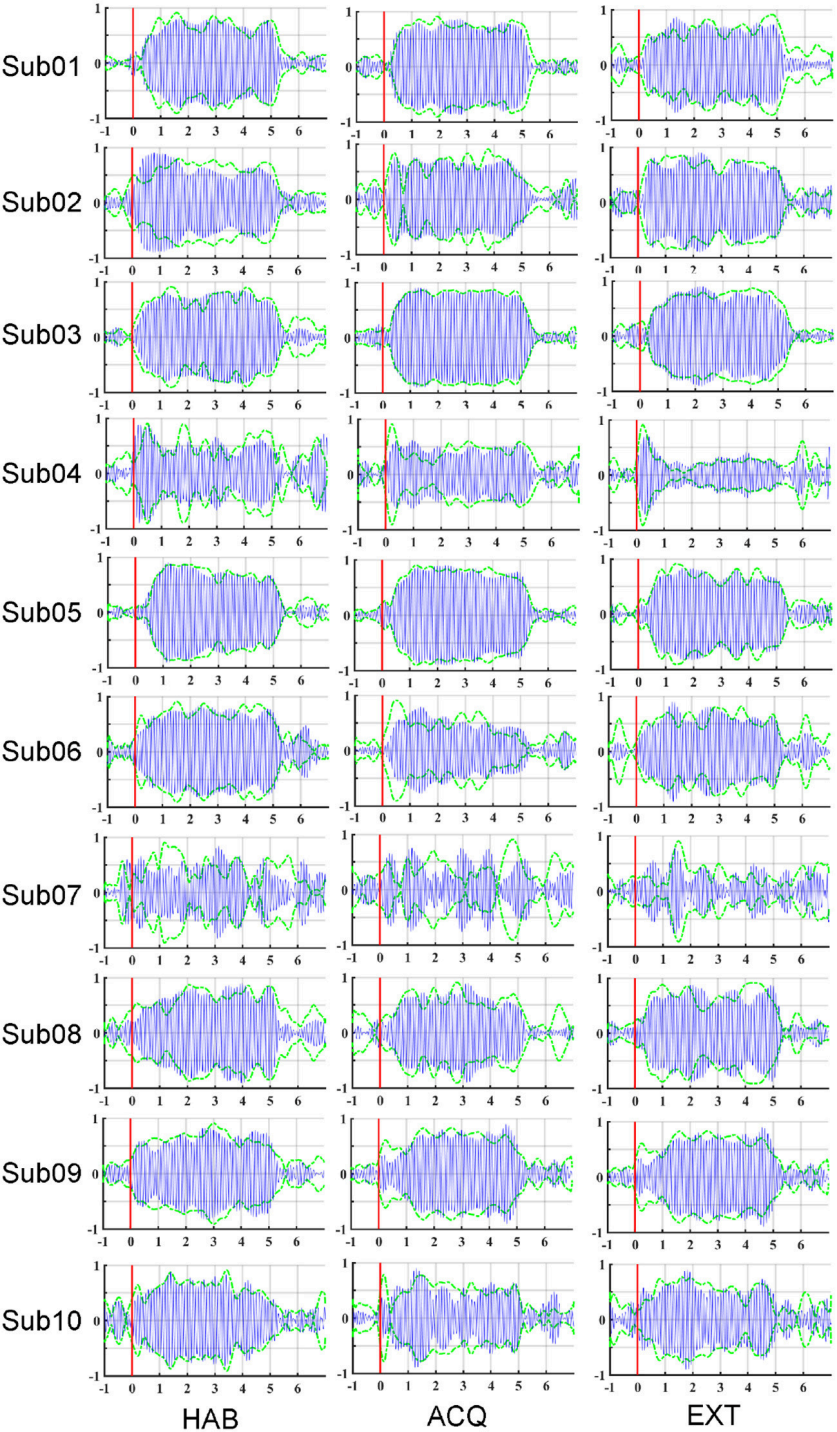
Maps of the averaged ssVEP epochs recovered by FSS (blue oscillations) along with the envelope of ssVEP optimized by ICA (dashed green line), for each participant (row) and experimental phase (column): habituation (HAB), acquisition (ACQ), and extinction (EXT). Stimulus-averaged epochs in the −1 to 7 s time period, with pre-stimulus −1 to 0 s and post-stimulus 0–7 s.

To illustrate the effects of preprocessing method, time course data of the averaged ssVEP epochs recovered by FSS across trials (blue traces) are overlapped with the results of ICA (dashed green traces) in Figure 3. The outline (equivalent to the envelope) of the blue oscillations (FSS-ssVEP) overall shows more stability and less noise across time, including in the baseline segment, compared to the green (ICA) envelope, which tends to be larger in the baseline segment and shows more temporal variation during the steady-state segment. Note that data from all phases were used in the joint optimization process. This will in some cases yield a less favorable signal-to-noise ratio compared to identifying optimal components for each single phase. However, a joint optimization across phases is expected to achieve more robust and more externally valid results, being based on more trials. On the other hand, noise such as alpha oscillations (generally in the range 8–13 Hz), or the absence of an ssVEP signal, may interfere with the optimization procedure, producing divergent results as shown for participant number 07.

### 3.2. ssVEP-BOLD Fusion Results

**Figure 4:**
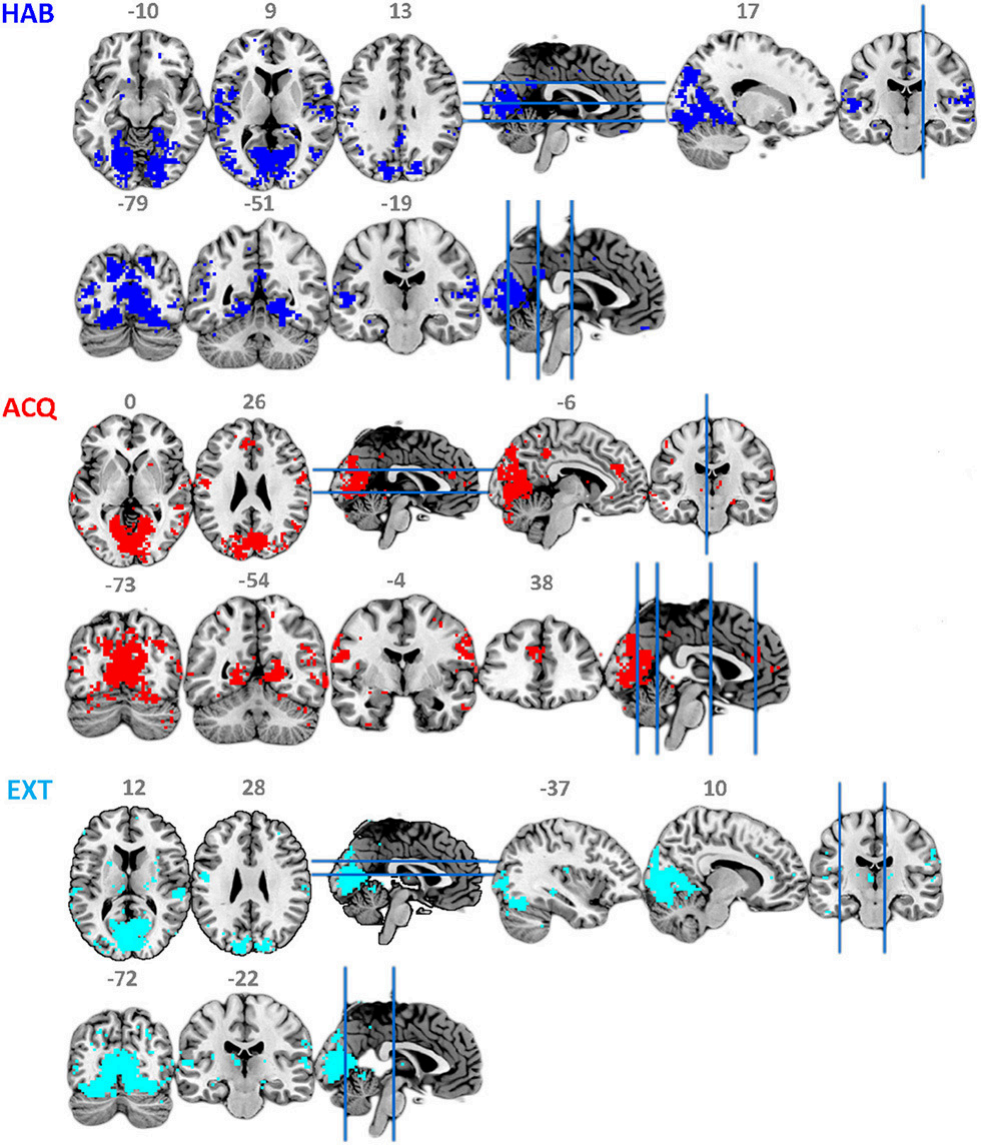
Maps of binomial test for FSS-ssVEP and BOLD fusion at each experimental phase: habituation (HAB), acquisition (ACQ), and extinction (EXT), threshold at 0.5.

Figure 4 and Table 2 show the ssVEP-BOLD co-variation with a standard 4 s delay. For each participant, voxels that survived individual permutation-controlled thresholding were kept and submitted to a group-level binomial test. As expected, the ssVEP-BOLD correlation map contained extended visual cortical areas, including the calcarine fissure, cuneus, occipital gyrus, and fusiform gyrus. Additional areas of ssVEP-BOLD co-variation were seen in the postcentral cortex, the rolandic operculum, and superior temporal gyrus. During the acquisition phase in which aversive learning occurs, we observed additional correlations between ssVEP and BOLD in the anterior cingulate cortex (ACC), precuneus, as well as the middle and superior temporal gyrus. Note that small but robust correlations between electrophysiological and hemodynamic measures during the same process have been consistently reported across different species, including human beings (see e.g., Boynton, 2011), for a discussion of this problem.

**Table 2:** Results for time-series based sVEP-BOLD correlation analysis.

**Location**	**Cluster size**	**CC**	**MNI**
	**(voxels)**	**(Max)**	**Coordinates**
L Calcarine	327	0.188	−9 −82 2
R Calcarine	303	0.184	15 −64 5
L Lingual	267	0.185	−6 −73 2
R Lingual	299	0.182	9 −64 5
L Middle occipital	47	0.124	−21 −91 20
R Middle occipital	144	0.160	33 −85 14
L Cuneus	224	0.193	−9 −82 20
R Cuneus	207	0.170	3 −73 20
L Middle Temporal	60	0.136	−60 −61 −1
R Middle Temporal	156	0.134	54 −64 2
R Superior temporal	39	0.126	54 −58 23
L Postcentral	135	0.166	−57 −4 20
R Postcentral	90	0.147	60 −1 35
L Precuneus	46	0.115	0 −46 41
R Precuneus	55	0.120	3 −67 23
L Fusiform	21	0.129	−21 −85 −16
R Fusiform	18	0.133	27 −67 −13
R Rolandic	23	0.137	63 −1 11
ACC	37	0.104	0 26 23

*MNI Coordinates, the absolute of correlation coefficient (CC) of correlation peaks are reported. R, Right; L, Left*.

**Figure 5:**
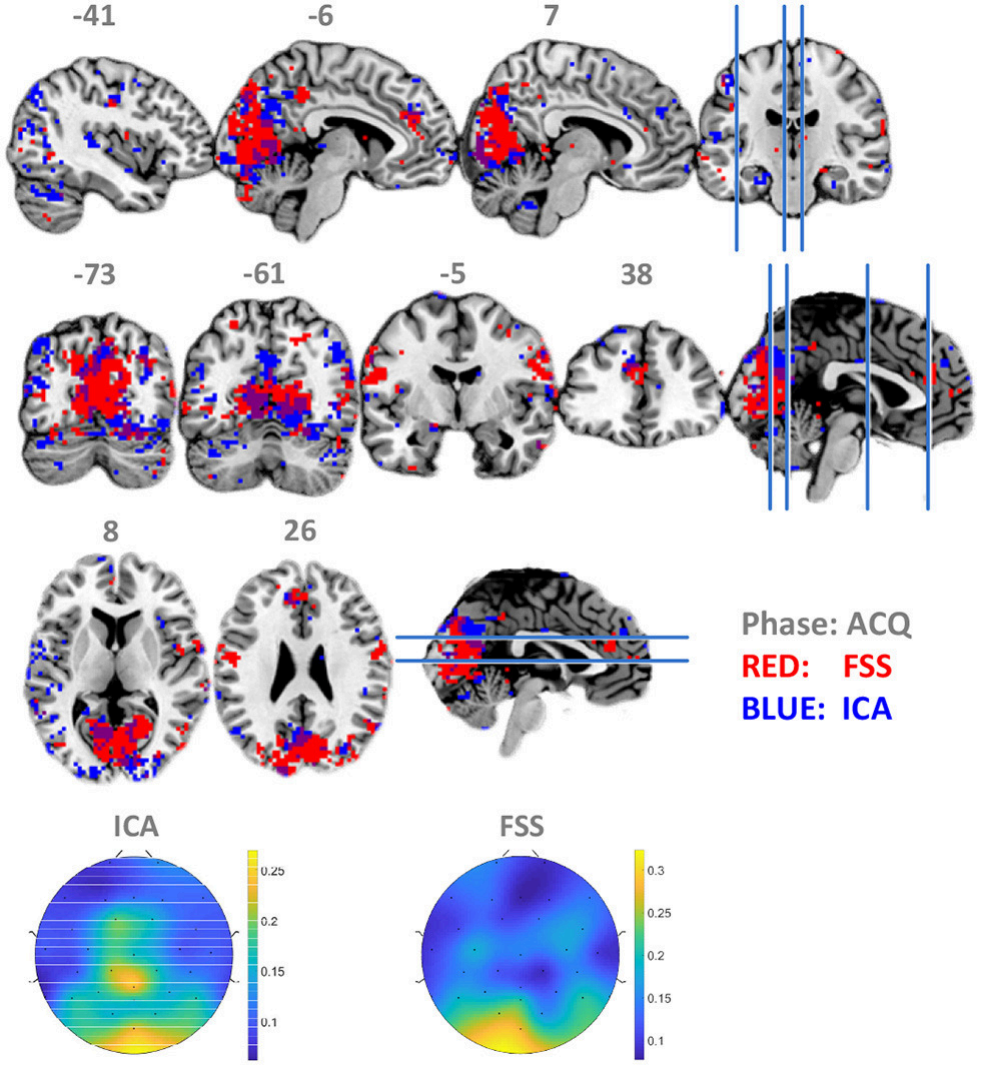
Maps of binomial test result for FSS-ssVEP and BOLD (red) in contrast to the ICA-ssVEP and BOLD (blue), acquisition phase (ACQ). The scalp topographical distribution of FSS weights are compared with ICA.

To establish that using the timing information contributes to suppressing noise and increase the robustness and validity of the fusion result, we set λ to zero so that the contrast function was only driven by independency term *J*, allowing the ICA to use data from all three phases equally for optimization by simulated annealing. Contrasting the spatial distribution of ssVEP-fMRI correlation maps between FSS and ICA, as shown in Figure 5, that ICA yielded a less coherent, and overall noisier spatial pattern, with fewer voxels in visual areas displaying the expected effect. In addition, when comparing the scalp topographical distribution of FSS weights with ICA weights, the FSS with the focal occipital topography is physiologically more plausible than the distributed ICA topography.

## 4. Discussion

The present study aimed to illustrate the usefulness of FSS as a preprocessing step for joint analyses of fMRI-BOLD and ssVEPs, strong oscillatory signals originating in visual cortex and modulated by re-entrant inputs from a number of anterior structures (Keil et al., 2009; Norcia et al., 2015). We used data from an experiment in which participants were aversively conditioned to associate the orientation of a phase-reversing grating with a mild electric shock (Petro et al., 2017). The experiment contained three distinct phases, which were used here to examine the convergence of ssVEP-BOLD covariation maps across three re-tests, leveraging the within-participant design of this study. We found substantial overlap of ssVEP-fMRI co-variation maps in extended visual cortex, superior temporal cortex, and parietal cortex. This is consistent with the sources of ssVEP established through intracranial recordings in humans and experimental animals (see e.g., Wittevrongel et al., 2018). Furthermore, the cross-participant and re-test convergence along with the large spatial extent of the ssVEP-fMRI covariation in visual cortex observed in the present study exceeds earlier analyses of this same data-set with different methods, i.e., parametric modulation analysis as implemented in SPM (Petro et al., 2017), and multivariate correlation analyses without the FSS preprocessing step (Ji et al., 2018). Because the covariation of both signal modalities across the time series in visual cortex can be considered a gold standard for analytical strategies, the fact that ssVEP-fMRI correlations were observed reliably in extended visual cortex suggests that FSS represents a desirable preprocessing step, positively affecting the validity of EEG-fMRI fusion.

Although no explicit comparisons between experimental phases were made, the ssVEP-fMRI co-variation map during the acquisition phase, in which the visual cues were occasionally paired with electric shocks, showed co-variation of ssVEP amplitude and BOLD signal in additional parietal and anterior structures. These structures included the anterior cingulate gyrus, known to be involved in fear acquisition and associative learning in general (e.g., Sehlmeyer et al., 2009; Fullana et al., 2016). As such, this finding further supports the validity of FSS-based preprocessing for ssVEP-fMRI fusion.

It should be noted that this study was limited in terms of sample size and in terms of signal-to-noise, low in some participants, despite the precise definition of ssVEPs in the frequency domain (Norcia et al., 2015). Although information theoretic approaches (Ostwald et al., 2010, 2011) have been proposed, which apply information criteria such as mutual information to quantify the association of multimodal signals, it is difficult to estimate the probability distribution reliably with small sample size, and the results are sensitive to free parameters. Furthermore, it is not within the scope of this manuscript to quantitatively compare many alternative pipelines to establish the benefits of FSS relative to other methods. Such analyses have been performed with other data types (Porcaro et al., 2010). However, both ongoing and future research will establish and further optimize preprocessing steps for ssVEP-fMRI fusion.

## 5. Conclusions

In conclusion, the present study demonstrated that FSS, in conjunction with a multivariate correlation and feature selection approach proposed previously (Ji et al., 2018), yields robust ssVEP-fMRI co-variation maps with high face validity (i.e., high correspondence between BOLD and ssVEP in visual cortex, where strong responses are expected to during visual stimulation) and with high replicability across re-tests (i.e., across three experimental phases) in the same participants. The work is important to neurorobotics for several reasons: (1) This approach could be used to examine a wide range of hypotheses regarding the neurophysiology of visual cortex and its function for biological and artificial systems, (2) it provides a means to conduct the multi-modal information fusion and correlation analysis to recover the underlying neurophysiological mechanisms, and (3) the core idea of the approach are inspiring for other brain data processing and the knowledge can be transferred to other applications as well.

## Ethics Statement

All procedures were approved by the institutional review board of the University of Florida and were consistent with the Declaration of Helsinki on studies with human participants.

## Author Contributions

All authors had full access to all the data in the study and take responsibility for the integrity of the data and the accuracy of the data analysis. AK and HJ: conceptualization. HJ, ZY, and AK: methodology. HJ and NP: investigation. NP: formal analysis. HJ, AK, and BC: writing. BC and AK: supervision. BC, NZ, and AK: funding acquisition.

### Conflict of Interest Statement

The authors declare that the research was conducted in the absence of any commercial or financial relationships that could be construed as a potential conflict of interest.
